# Books, Good and Otherwise

**Published:** 1887-01-22

**Authors:** 


### Books, Good and Otherwise.

*Juno, a Hunting Story.* A Complete Novel, by a New Author. No. 19. *Monthly Magazine of Fiction.* London: Stevens, 421, Strand.

The story of *Juno* is not, strictly speaking, a "hunting narrative" as the title might lead us to suppose; it is more truly a story—short, pathetic, and passionate, dealing with the old theme of woman's constancy and man's desertion. The character of the heroine is a weird and fascinating one, and, for the penalty paid by the hero for his passing fancy for the beautiful Roona Malone, we must refer the reader to the pages of *Juno.* On a railway journey, or for a quiet half-hour when the day's work is over, no more interesting companion than *Juno* could be found—so full of light and shade; without dulness or vapidity; instinct with incident and life—it is yet absolutely free from any of the objectionable elements so often found in works of fiction, and it has the merit of not being too long.

*Home Rule "Wrinkles" for Ladies.* By Aunt Betsy. London: Swan Sonnenschein and Co., Paternoster Row.

In *Home Rule "Wrinkles" for Ladies,* "Aunt Betsy" offers some sensible suggestions for the maintenance of "Home Rule" by the fair sex on its best basis. To young married ladies, these "wrinkles" will be of great assistance in many little difficulties which are sure to confront them in the early days of housekeeping; and not alone to those used to the art will this little book be of use; but to many others who have the care of children and servants, Aunt Betsy's hints will be invaluable. On all matters connected with woman's "special sphere"—servants, children, housekeeping, dress, toilet, nursery, and other matters of equal interest,—Aunt Betsy has a few words of sensible advice and direction to give. We recommend this little handbook to the notice of all ladies interested in the cultivation of domestic "wrinkles."

We have received the following books for review :—

MACMILLAN & CO.
Special Pathological Anatomy. By D. Macalister.
STEVENS, W.
Juno : a Hunting Story.
STANFORD, E.
Transactions of the Sanitary Institute of Great Britain. Vol. VII.
SWAN SONNENSCHEIN & CO.
Home Rule Wrinkles. By Aunt Betsy.

EXCHANGES.

CHURCHILL, J. & A.
Journal of Mental Science. April, July, and Oct. 1886, and Jan. 1887.
HATCHARDS.
Friendly Work. May 1885, and April 1886.

